# Measuring motivation for medical treatment: confirming the factor structure of the Achievement Motivation Index for Medical Treatment (AMI-MeT)

**DOI:** 10.1186/s12911-016-0260-0

**Published:** 2016-02-19

**Authors:** Taichi Hatta, Keiichi Naria, Kazuhiro Yanagihara, Hiroshi Ishiguro, Toshinori Murayama, Masayuki Yokode

**Affiliations:** Uehiro Research Division for iPS Cell Ethics, Center for iPS Cell Research and Application (CiRA), Kyoto University, 53 Shogoin-Kawahara-cho, Sakyo-ku, 606-8507 Kyoto Japan; Clinical Innovative Medicine, Institute for Advancement of Clinical and Translational Science (iACT), Kyoto University Hospital, 54 Shogoin-Kawahara-cho, Sakyo-ku, 606-8507 Kyoto Japan; Department of Medical Oncology, Kansai Electric Power Hospital, 2-1-7 Fukushima, Fukushima-ku, 553-0003 Osaka Japan; Department of Target Therapy Oncology, Graduate School of Medicine, Kyoto University, 54 Shogoin-Kawahara-cho, Sakyo-ku, 606-8507 Kyoto Japan; Department of Clinical Development, Kanazawa University Hospital, 13-1 Takara-machi, 920-8641 Kanazawa, Japan

**Keywords:** Cancer treatment, Patients’ motivation, Self-construal, Structural equation modeling, Informed consent, Japan

## Abstract

**Background:**

Developments in chemotherapy have led to changes in cancer care in Japan, with the government promoting a transition to outpatient chemotherapy. This requires patients and their families to participate more actively in treatment than in the past. However, it remains unclear how patients’ motivation for medical treatment affects clinical consultations with their physicians. To investigate this, we developed a psychological index called the Achievement Motive Index for Medical Treatment (AMI-MeT), which comprises self-derived achievement motivation (AMS) and achievement motivation derived from others (AMO). However, its factor structure has not yet been confirmed in populations other than healthy university students. Thus, the aims of this study were to confirm the factor structure of the AMI-MeT in other groups and to determine the convergent and divergent validity of the AMI-MeT.

**Methods:**

The AMI-MeT was administered to university students (*n* = 414), apparently healthy workers (*n* = 154), and cancer patients (*n* = 51). Multi-group confirmatory factor analysis was conducted and the mean scores of the AMI-MeT were compared between the groups. Correlations between the AMI-MeT and the Self-Construal Scale, comprising independent self-construal (IndSC) and interdependent self-construal (InterSC) subscales, were investigated in another group of students (*n* = 335).

**Results:**

The multi-group confirmatory factor analysis supported a two-factor structure of the AMI-MeT: the weak invariance model was the best fit for the data. The mean scores of the AMI-MeT in apparently healthy workers and cancer patients were significantly higher than that in students (*P* < .01). The correlation analysis revealed that AMS scores were associated with IndSC scores (*r* = .25, *P* < .01) and AMO scores with InterSC scores (*r* = .30, *P* < .01).

**Conclusion:**

The two-factor model of the AMI-MeT was deemed appropriate for all three groups, and the subscales of the AMI-MeT successfully reflected the self and other dimensions. The AMI-MeT appears to be an effective tool for measuring medical treatment motivation, making it useful in participant observational research on medical consultations for Japanese cancer treatment.

**Electronic supplementary material:**

The online version of this article (doi:10.1186/s12911-016-0260-0) contains supplementary material, which is available to authorized users.

## Background

Japan has a history of withholding cancer notification [1], but many surveys have indicated a gradual shift towards disclosing such information [2–7]. Furthermore, developments in chemotherapy have led to changes in cancer care in Japan, and the government has promoted a transition towards outpatient chemotherapy since 2007 [8]. Cancer patients and their families have acknowledged the benefits of outpatient chemotherapy; however, it also requires them to participate more actively in treatment than in the past. Despite much research on Japanese cancer patients’ preferences for receiving bad news [9–11], it remains unclear how patients’ motivation to obtain medical treatment affects clinical consultations with their physicians. To investigate this, we launched the Mixed-methods Observational Research for Informed Consent (MORE-IC) project [12, 13], wherein we performed quantitative and qualitative participant observation of informed consent consultations involving cancer patients. However, this project demanded the availability of a convenient/easily answerable questionnaire for quantifying patients’ interest in medical treatment before starting their consultations. As no such tool exists, we developed a psychological index of patient motivation for treatment, called the Achievement Motive Index for Medical Treatment (AMI-MeT).

Although many patients have been inclined to actively participate in treatment decision making in Japan [2], those who actually can clearly convey their intentions to their physicians remain a relative minority. Physician–patient communication, and the intention underlying patients’ communication about their medical treatment, relates to several factors, such as the social values of the hospital, expectations for medical treatment, preferences for communication style, and so on. Notably, these factors can be interpreted according to the theory of planned behavior [14], as they correspond to the concepts of attitude toward the behavior, subjective norms, and perceived behavior control, which all influence intention. Given that few patients can clearly convey their intentions, we thought it necessary to include the potential interest behind the intention for medical treatment in the concept of motivation for medical treatment. On the other hand, the dualistic concept of achievement motivation in the AMI-MeT was derived from the concepts of self-fulfillment and competition achievement [15], which are analogous to the concepts of self-achievement [16] and ordinal achievement motivation [17, 18], respectively. According to literature on patients’ narratives [19–21], patients often seek to remain themselves during long-term treatment and to create a new identity or continuity of self at the end of life; thus, we supposed that the process of medical decision making can be construed as a form of self-actualization [16] or individuation [22]. To understand how patients view medical treatment, we also referred to the other dualistic concepts of the self and motivation: construals of the self as independent/interdependent [23], and intrinsic/extrinsic motivation [24]. Using these concepts, we holistically defined achievement motivation for medical treatment as a personal interest and value that could exert a potent influence on medical decision making and would be influenced by social norms or expectations.

In an earlier study, we conducted an exploratory factor analysis to derive the subscales of the AMI-MeT, which were as follows: self-derived achievement motivation (AMS), containing 5 items (e.g., “I want to make the best decision for me,” “Even if several therapies are available, I want to find the one that’s preferable for me”); and achievement motivation derived from others (AMO), also containing 5 items (e.g., “It’s important to strive for advanced medical care,” “I want to be treated at a noted hospital”) (Table 1) [25]. The scale items are rated on a 7-point Likert-type scale ranging from 1 (*strongly disagree*) to 7 (*strongly agree*). In the previous pilot study, these two subscales had adequate reliability (Cronbach’s alpha = .78–.86), and the construct validity of the AMI-MeT was confirmed through a comparison with Horino’s achievement motive scale [15]. The items of the AMI-MeT were developed in Japanese and translated into English for the present paper (Table 1). As shown in Table 1, the items do not directly represent motivation itself, but indicate a personal interest and value for medical treatment, because of the broad definition of motivation used in this study.

**Table 1:** A list of items on the Achievement Motive Index for Medical Treatment

Sentence of each items
Achievement Motivation Derived from Other (AMO)
Q1: It’s important to strive for advanced medical care. Q2: I want to be treated at a noted hospital. Q3: Selecting a therapy grounded in medicine is important. Q4: I’d be happy to receive better-than-average care. Q5 If treatment works well, that means living longer.
Self-derived Achievement Motivation (AMS)
Q6: I want to make the best decision for me. Q7: I should be convinced with various things that matter. Q8: It’s important for me to follow my heart rather than to trouble myself with the ideas of others. Q9: Even if several therapies are available, I want to find the one that’s preferable for me. Q10: I want to express my wishes, even if it’s something that’s not very significant.

However, because the AMI-MeT was preliminarily developed in a rather small sample of university students, its reliability and validity remain insufficient. Specifically, the AMI-MeT was tested only using exploratory factor analysis in a single sample of university students; no confirmatory factor analysis has been performed in other populations. Additionally, the AMI-MeT has not yet been applied to the patient population—so far, it can only assess the motivation of university students, who do not have the same needs or interests as do patients. Finally, it remains unclear whether the subscales (AMS and AMO) are in fact based on dualistic concepts of the self, since they have been found to correlate only with achievement for self-fulfillment/competition [15]. These issues indicated the necessity for further investigation of the AMI-MeT among university students as well as other populations.

The aims of this article are, in two studies, to demonstrate that the AMI-MeT data from university students, apparently healthy workers, and cancer patients fit the two-factor model; that AMI-MeT scores differ between the three groups; and that the AMI-MeT subscales correlate with dualistic concepts of the self.

## Methods

### Ethical considerations

The questionnaire surveys were approved by the institutional review board at Kyoto University Graduate School of Medicine (E-570, E-1107, E-1254, E-1387, and E 1426) and the Kyoto Industrial Health Association (April 2011).

### Study 1: investigating the factor structure and the mean scores of the AMI-MeT in multiple groups

To confirm the factor structure of the AMI-MeT in more diverse groups and demonstrate that it accurately assesses motivation for medical treatment, we administered the AMI-MeT to several groups with varying needs for medical treatment: university students, apparently healthy workers, and cancer patients. It is likely that most university students, a young and comparatively healthy population, are less likely to need medicine or to go to the hospital, making them generally less motivated to do. In contrast, cancer patients who have already been diagnosed and informed of their diagnosis might feel a greater need for medical treatment—therefore, they are perhaps a relatively highly motivated population. Workers who had come to a medical institution for a health check were proposed to be a middle population in terms of motivation, somewhere between university students and cancer patients, as they would be potentially aware of the concerns or needs for medical treatment, but not to the same extent as would cancer patients. In other words, given that this group had voluntarily gone for a health screening, they were likely concerned about having undiagnosed diseases or perhaps realized the necessity of obtaining treatment. As such, we defined them as an “apparently healthy” group.

#### Participants and measure

The first group comprised university students from the Kansai region of Japan, who were asked to complete the questionnaire after a psychology class between October and November 2011. Of the 456 students given the questionnaire, 414 responded to all items of the questionnaire (317 women and 97 men; mean age = 20.8; *SD* = 5.4); we excluded any answer sheets that contained missing data. The second group comprised apparently healthy workers—that is, workers who had not yet been diagnosed with a disease but who had voluntarily undergone a health screening at a medical institution of the Kyoto Industrial Health Association between April 2011 and March 2012. Two hundred and thirty-three workers received the questionnaire after their health checks at the institution, and 164 completed it; of these, 154 valid responses were obtained (51 women and 103 men; mean age = 46.3; *SD* = 12.4). The third group comprised patients who had been diagnosed with breast or lung cancer and had come to start chemotherapy at Kyoto University Hospital between January and July 2009 or between April 2012 and January 2013. The questionnaire survey was administered to cancer patients who were part of the MORE-IC project. After the first author explained the aim of the MORE-IC project, 51 valid responses were obtained (32 women and 19 men; mean age = 60.8; *SD* = 11.6).

Motivation for medical treatment was measured using the AMI-MeT, as described previously.

#### Analysis 1: multi-group structural equation modeling

To confirm the factor structure of the AMI-MeT in all three groups, we performed a confirmatory factor analysis with maximum likelihood estimation. Specifically, we used structural equation modeling (SEM) with a multi-group procedure [26–28] and compared the model invariance: configural, weak, strong, and complete invariance. Configural invariance is the simplest form of invariance, wherein no constraints are placed on the parameters; it is satisfied if a defined factor structure is a good fit to the data for all groups. Then, weak, strong, and complete forms of invariance were tested in sequence by placing constraints on the parameters (i.e., factor loadings, factor variances/covariance, and error variances) of the configural invariance model [27, 28]. Specifically, testing weak invariance involves constraining the factor loadings to be equal between the groups; when weak invariance is satisfied (i.e., the model is a good fit), the latent variables being measured are the same across the groups. Testing strong invariance involves placing equality constraints on both the factor loadings and the factor variances/covariance between groups; if it is satisfied, group comparisons of the domain means can be performed. Complete invariance holds if the factor loadings, factor variances/covariance, and error variances are equivalent for the groups [28].

To evaluate and compare these models, the chi-squared model test (χ^2^/df) and chi-square difference test (Δχ^2^) [29] were conducted. To test model fit, we used the following fit indices: the goodness of fit index (GFI), the adjusted goodness of fit index (AGFI), the comparative fit index (CFI), the Akaike information criterion (AIC), and the root mean square error of approximation (RMSEA). The GFI, AGFI, and CFI range between zero (0) and unity (1), with unity meaning a perfect fit to the data. The AIC adjusts for model complexity [30], and smaller values indicate better fit [31]. An RMSEA of about 0.05 or less indicates a close fit of the model in relation to the degrees of freedom, while about 0.08 or less indicates a reasonable error of approximation. However, such cut-offs should be regarded as guidelines rather than as “golden rules” when assessing model fit [32]. The confirmatory factor analysis was performed with AMOS 22.0. The chi-square difference test [29] and the internal consistency reliability coefficient McDonald’s ω [33–36] were calculated with Microsoft Excel.

#### Analysis 2: one-way analysis of variance of AMI-MeT scores between the three groups

To demonstrate whether the AMI-MeT score (ten items) of cancer patients and workers are higher than are those of students, we performed a one-way analysis of variance (ANOVA with post hoc pairwise Bonferroni-adjusted comparisons) between the three groups. The statistical analysis was performed with SPSS 22.0. The effect size (η^2^) was also calculated with Microsoft Excel.

### Study 2: correlating the subscales of the AMI-MeT with the dualistic concept of the self

To determine the convergent and divergent validity of the AMI-MeT, we examined whether it correlates with another theoretically relevant psychological scale. In this case, we referred to the concept of self-construal [23], which is typically defined as how individuals see the self in relation to others [37]. Markus and Kitayama coined the term “self-construal” in describing differences in the ways that Americans and Japanese define and make meaning of the self; they identified two types of self-construal: independent and interdependent [23]. Cross et al. reviewed the two types of self-construals, as follows:

Markus and Kitayama (1991) proposed that Europeans and Americans construe the self as fundamentally individual and separate from others, and they labeled this the independent self-construal (IndSC)…. In contrast, Markus and Kitayama (1991) pointed out that the Japanese tend to construe the self as fundamentally connected to others and defined by relationships with others, which they labeled the interdependent self-construal (InterSC). ([37] p. 143)

In later works, InterSC was proposed to consist of two components: “relational component” and “group-oriented component” [37–39]. Although Markus and Kitayama (1991) defined InterSC in terms of close relationships and important in-groups, “the connection between IndSC and individualism and between InterSC and collectivism is clear—so clear, in fact, that it can be difficult to distinguish between self-construal and individualism–collectivism” ([37] p. 143).

Given this conceptual background, we assumed that AMS (which includes items such as “I want to make the best decision for me” and “Even if several therapies are available, I want to find the one that’s preferable for me”) would be correlated with IndSC; in other words, those who fundamentally construe themselves as individuals may take a more personal interest in medical decision making. We also supposed that AMO (e.g., “It’s important to strive for advanced medical care” and “I want to be treated at a noted hospital”) would show a correlation with InterSC; that is, having a group-oriented awareness of medicine could affect the value individual respondents place on medical treatment that is based on social norms or expectations.

#### Participants and measure

The questionnaire was distributed to university students in the Kansai region of Japan. They were asked to complete the questionnaire after a psychology class in May 2012. The data from Study 2 was part of another questionnaire survey. Notably, some of the respondents from Study 2 could have answered the questionnaire in Study 1. To avoid analyzing the same students twice, we made sure to treat them as separate groups while handing out the questionnaire. Of the 518 students given the questionnaire, 335 filled it in completely (200 women and 135 men; mean age = 19.6; *SD* = 2.9); we excluded any answer sheets that contained missing data.

Motivation for medical treatment was measured using the AMI-MeT. To validate the dualistic concepts of the AMI-MeT subscales, we administered the Self-Construal Scale (SCS) [40], which is based on the concept of self-construal [23]. The SCS was translated into Japanese by Takahashi et al. [41]. Using a 7-point Likert-type scale (1 = *strongly disagree* to 7 = *strongly agree*), participants indicated their level of agreement with 12 items assessing IndSC (e.g., “I prefer to be direct and forthright when dealing with people I’ve just met,” “I enjoy being unique and different from others in many respects”) and 12 statements assessing InterSC (e.g., “It is important for me to maintain harmony within my group,” “It is important to me to respect decisions made by the group”).

#### Analysis 3: correlations between the subscales of the AMI-MeT and SCS

To demonstrate the convergent and divergent validity of the subscales of the AMI-MeT with the dualistic concepts of the self, we calculated the Pearson’s correlation coefficients between the AMI-MeT subscales and SCS subscales. This statistical analysis was performed with SPSS 22.0.

## Results

### Results from analysis 1: multi-group structural equation modeling

**Fig. 1:**
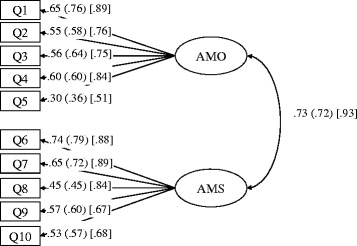
The standardized values in the hypothesized weak invariance model. The circles represent the latent factors of AMO and AMS, the rectangles represent measured indicators (i.e., Q1–Q5 represent AMO, and Q6–Q10 represent AMS), the lines connecting latent factors to indicators represent factor loadings, and the curve connecting the two latent factors represents covariation. The numbers provided are standardized values for the student group, the worker group (in parentheses), and the patient group [in brackets]

Descriptive statistics and correlation matrixes of the AMI-MeT items are reported in detail in Additional file 1. The results of the multi-group SEM with maximum likelihood estimation supported the two-factor structure of the AMI-MeT. The configural invariance model fit the data for both groups (CFI: .911; GFI: .929; AGFI: .886; RMSEA: .048). After testing the configural invariance model, we tested the other invariance models according to several sets of constraints. The chi-square test (χ^2^/df) and the chi-square difference test (Δχ^2^) showed that the weak invariance model was the best fit for the data (Table 2), although the items did not follow a normal distribution (Additional file 1). The fit indices also indicated that the weak invariance model was the best fit for the data (Table 2). Accordingly, we regarded the weak invariance model as the best model, and have displayed the standardized estimations of the model in Fig. 1. In the weak invariance model, the obtained ω for AMO and AMS ranged from .67–.75 and .72–.79, respectively, indicating high internal consistency. The results of the SEM and the high internal consistency reliability coefficients indicate that the two-factor model of the AMI-MeT was reliable and that the factor loadings of the AMO and AMS were common across the three groups.

**Table 2:** Test of measurement equivalences for Achievement Motive Index for Medical Treatment

Fit indexes	χ2	df	p	χ^2^/df	Δχ^2^	Δdf	p’	CFI	GFI	AGFI	AIC	RMSEA
Configural invariance model	244.051	102	.000	2.393	-	-	-	.911	.929	.886	370.051	.048
Weak invariance model	265.043	118	.000	2.246	20.992	16	.179	.908	.923	.893	359.043	.045
Strong invariance model	301.086	120	.000	2.509	57.035	18	.000	.887	.912	.878	391.086	.049
Complete invariance model	400.484	140	.000	2.861	156.433	38	.000	.837	.888	.868	450.484	.055

Equivalences for AMI-Met were tested in sequence by placing constraints on the parameters (i.e., factor loadings, factor variances/covariance, and error variances). Configural invariance is the simplest form without any constraints on the parameters. Weak invariance constrains the factor loadings; strong invariance constrains the factor loadings and factor variances/covariance; and complete invariance constrains the factor loadings, factor variances/covariance, and error. The difference in the χ^2^ values (Δχ^2^) and the degrees of freedom (Δdf) were calculated as follows: *Δχ* ^2^ = *χ* ^2^_nested model_ − *χ* ^2^_configural invariance model_, and *Δ*df = df_nested model_ − df_configural invariance model_. p’ indicates the significance distributed from Δχ^2^ and Δdf [24]

### Result from analysis 2: one-way analysis of variance of AMI-MeT scores between the three groups

The mean score of the AMI-MeT (ten items) was compared between the three groups. The score of workers and cancer patients were significantly higher than was that of students (*F*(2, 616) = 9.31, *P* < .01, η^2^ = .029; worker vs. student: *P* < .01; cancer patient vs. student: *P* < .01). The difference was also found for the mean scores of the AMO (five items); in contrast, AMS scores (five items) did not significantly differ between groups (Table 3).

**Table 3:** Scores of the Achievement Motive Index for Medical Treatment in Study 1

	Students		Workers		Cancer patients				Multiple comparison
	*n* = 414		*n* = 154		*n* = 51				
	Mean	S.D.	Mean	S.D.	Mean	S.D.	F	*p*	
AMI-MeT	55.35	6.44	56.89	5.94	59.33	10.99	9.31	.000	S^a^ < W^b^, P^c^
AMO	26.52	3.94	28.14	3.66	29.70	5.67	19.55	.000	S < W, P
AMS	28.83	3.53	28.75	3.22	29.67	5.83	1.30	.274	

^a^S = Student group

^b^W = Worker group

^c^P = Cancer patient group

### Result from analysis 3: correlations between the subscales of the AMI-MeT and SCS

Before examining the correlations, we calculated the Cronbach’s alphas of the subscales of both the AMI-MeT and the SCS, as follows: AMS and AMO, α = .79 and .75, respectively; IndSC and InterSC, α = .69 and .74, respectively. The correlation analysis revealed that the AMS scores were more related with the IndSC scores (*r* = .25, *P* < .01) than with the InterSC scores (*r* = .19, *P* < .01), and that the AMO scores were more related with the InterSC scores (*r* = .30, *P* < .01) than with the IndSC scores (*r* = .14, *P* < .05). Because there was a high correlation between AMS and AMO (*r* = .64, *P* < .01), a partial correlation analysis was also conducted. When we controlled for AMO in investigating the relationships between AMS and IndSC and between AMS and InterSC, we found partial correlations of *r* = .22 (*P* < .01) and *r* = .00 (*n.s.*), respectively. Furthermore, when we controlled for AMS in investigating the relationships between AMO and IndSC and between AMO and InterSC, we found partial correlations of *r* = -.03 (*n.s.*) and *r* = .24 *(P* < .01), respectively.

## Discussion

We examined the factorial validity and the internal consistency reliability of the AMI-MeT by confirming the two-factor model in multiple groups (Analysis 1), and examined the concurrent validity of the AMI-MeT, as it exhibited significant differences in the mean score between groups (Analysis 2). Additionally, we demonstrated the convergent and divergent validity of the subscales of the AMI-MeT using the SCS, as we believed that motivation for medical treatment would be theoretically linked with the concept of self-construal (Analysis 3).

As demonstrated in Analysis 1, the AMI-MeT has a two-factor structure (AMS and AMO). Notably, as shown in Analysis 2, the AMO scores of workers and cancer patients were significantly higher than were those of students, while the AMS scores did not differ among the groups. We expected both workers and cancer patients to recognize the need for medical treatment because they had actually come to the hospital, whereas university students might not recognize such a need—thus, the items of the AMO subscale may accurately reflect the value a respondent assigns on the basis of social norms or expectations, rather than personal interest. Accordingly, the AMO could be considered to have good concurrent validity. It must be noted that the concurrent validity of the AMS could not be discussed in the same manner as that of the AMO in this research setting, because the items of the AMS are not as reflective of visiting the hospital as are the items of the AMO.

Regarding the convergent and divergent validity of the subscales of the AMI-MeT (Analysis 3), the hypothesized strong associations (i.e., the correlations between AMS and IndSC and between AMO and InterSC, which were *r* = .25 and .30, respectively) were barely stronger than were those expected to be low (i.e., the correlations between AMS and InterSC and between AMO and IndSC, which were *r* = .19 and .14, respectively). However, given that the conceptual similarity between AMS and AMO could result in their being highly correlated (which was supported by our results; *r* = .64, *P* < .01), we also conducted a partial correlation analysis to eliminate the potential confounds. As the partial correlation analysis indicated, there were weak but significant associations between AMS and IndSC and between AMO and InterSC (.22 and .24, respectively), and no associations between AMS and InterSC and between AMO and IndSC (.00 and -.03, respectively). Thus, this partial correlation analysis while controlling for AMS and AMO supports the convergent and divergent validity of the subscales of the AMI-MeT.

With regard to the patient group, our interpretations must be carefully made; if a patient would urgently require medication and clearly express his or her interest, it would seem natural for him or her to mark the highest score on every question of the AMI-MeT. As such, at this point, it appears difficult to divide patients’ motivation into personal interest or established value based on social norms and expectations using the AMI-MeT. Additionally, AMO and AMS scores showed ceiling effects for cancer patients as well as stronger correlations in this group. An explanation for these results could be that cancer patients urgently require medication and hence have a high interest in treatment. As such, this ten-item AMI-MeT would be useful in the field of medical decision making for detecting patients with lower levels of motivation, who may require further assessment and support.

### Application of the AMI-MeT in future analyses

To identify how motivation functions in this respect and investigate the qualities of actual physician–patient interaction during clinical consultation, we created the MORE-IC Project [12, 13]. In this study project, we collected both quantitative and qualitative data, including AMI-MeT scores, audio records, and field notes of participant observations of informed consent consultations for starting chemotherapy. As the results of the present study showed, the AMI-MeT can accurately distinguish patients with high and low motivations, and provides baseline data on patient motivation for our research project. In the future, the AMI-MeT will allow formal comparison of the process of informed consent consultations between patients with high and those with low motivation.

### Limitations

The limitations are as follows. First, the two-factor model of the AMI-MeT is incomplete. Due to an unbalanced sample size, the results of the SEM were biased by the data from the student group. If each group were equal in sample size, several fit indexes could be changed and it is possible that the two-factor model would not have been proposed. Given that cancer patients showed higher correlations between AMS and AMO than did the other groups, the AMI-MeT could have a one-factor model. A one-factor model would not precisely contradict our results herein, because our two-factor model does not specify that the AMO and AMS subscales were independent. Thus, the factorial validity of the AMI-MeT remains to be clarified.

Second, although the results of the correlation analysis demonstrated the convergent and divergent validity of AMI-MeT subscales with the SCS subscales, the correlations between AMS and IndSC and between AMO and InterSC must be interpreted cautiously for the following two reasons. First, the Cronbach’s alphas of the IndSC and InterSC subscales seemed to be lower (α = .69 and .74, respectively) than in previous studies. Takahashi et al. (2009) reported Cronbach’s alphas of .48–.58 for the IndSC subscale and .86–.87 for the InterSC subscale [41]; overall, the inter-item reliabilities of these two subscales tend to be adequate at best (α = .73–.74 and .69–.70 on the IndSC and InterSC subscales, respectively [40]). Second, there was a conceptual mismatch between the AMO and InterSC subscales. That is, the items of the AMO subscale did not refer to the relationship with clinical professionals but to the collective awareness for medical treatment; in contrast, Markus and Kitayama defined InterSC according to relationships with others [23]. These reasons doubtlessly limit the convergent and divergent validity of AMI-MeT subscales in this research.

Finally, our results cannot generalize to all cancer patients. We limited our sample to cancer patients who were diagnosed with breast or lung cancer and who were asked to answer the AMI-MeT before their clinical consultation for starting outpatient chemotherapy in a university hospital. Because individuals’ concerns about cancer treatment likely differ depending on their possible treatment options, disease stage, or social role, further investigation is required to explore how patients’ interests vary.

## Conclusion

We confirmed the validity and reliability of the two-factor model of the AMI-MeT and checked that the AMI-MeT could accurately measure motivation for medical treatment among different groups. In the future, when we investigate how shared decision making proceeds between Japanese physicians and patients or what occurs in their conversation process, the AMI-MeT will be useful tool for detecting patients with high and low motivation.

## Additional file

Additional file 1:
**The descriptive statistics and correlation matrix of the Achievement Motive Index for Medical Treatment.** (PDF 59 kb)

## Abbreviations

AGFI: adjusted goodness of fit index
AIC: Akaike information criterion
AMI-MeT: Achievement Motive Index for Medical Treatment
AMS: self-derived achievement motivation
AMO: achievement motivation derived from others
CFI: comparative fit index
GFI: goodness of fit index
IndSC: independent self-construal
InterSC: interdependent self-construal
RMSEA: root mean square error of approximation
SCS: Self-construal scale
SEM: structural equation modeling
