# Erratum to: Measuring motivation for medical treatment: confirming the factor structure of the Achievement Motivation Index for Medical Treatment (AMI-MeT)

**DOI:** 10.1186/s12911-016-0266-7

**Published:** 2016-03-02

**Authors:** Taichi Hatta, Keiichi Narita, Kazuhiro Yanagihara, Hiroshi Ishiguro, Toshinori Murayama, Masayuki Yokode

**Affiliations:** Uehiro Research Division for iPS Cell Ethics, Center for iPS Cell Research and Application (CiRA), Kyoto University, 53 Shogoin-Kawahara-cho, Sakyo-ku, 606-8507 Kyoto Japan; Clinical Innovative Medicine, Institute for Advancement of Clinical and Translational Science (iACT), Kyoto University Hospital, 54 Shogoin-Kawahara-cho, Sakyo-ku, 606-8507 Kyoto Japan; Department of Medical Oncology, Kansai Electric Power Hospital, 2-1-7 Fukushima, Fukushima-ku, 553-0003 Osaka Japan; Department of Target Therapy Oncology, Graduate School of Medicine, Kyoto University, 54 Shogoin-Kawahara-cho, Sakyo-ku, 606-8507 Kyoto Japan; Department of Clinical Development, Kanazawa University Hospital, 13-1 Takara-machi, 920-8641 Kanazawa, Japan

The original version of this article [1] contained an error. After publication it came to the authors’ attention that one of the authors’ names was incorrectly spelt. The original spelling reads as ‘Keiichi Naria’, however the correct spelling should be Keiichi Narita. This has now been updated on the BMC website.

## References

[1] Hatta T (2016). Measuring motivation for medical treatment: confirming the factor structure of the Achievement Motivation Index for Medical Treatment (AMI-MeT). BMC Med Informat Decis Making.

