# Supplementary material for: Measuring motivation for medical treatment: confirming the factor structure of the Achievement Motivation Index for Medical Treatment (AMI-MeT)
Source: BMC Med Inform Decis Mak. 2016 Feb 19;16:22. doi: 10.1186/s12911-016-0260-0 (PMC4759773; doi:10.1186/s12911-016-0260-0)
Supplement: Additional file 1: — The descriptive statistics and correlation matrix of the Achievement Motive Index for Medical Treatment. (PDF 59 kb) [file 12911_2016_260_MOESM1_ESM.pdf]

Additional file 1

The descriptive statistics and correlation matrix of the Achievement Motive Index for Medical Treatment.

Students (n = 414)

	M	SD	Skewness	Kurtosis	1.	2.	3.	4.	5.	6.	7.	8.	9.	10.
Q1	5.65	1.12	-0.57	-0.21										
Q2	5.79	1.02	-1.04	2.05	.258									
Q3	5.34	1.12	-0.30	-0.27	.381	.286								
Q4	5.38	1.32	-0.79	0.72	.448	.325	.324							
Q5	4.36	1.46	-0.18	-0.37	.236	.138	.208	.230						
Q6	6.10	0.96	-1.04	1.49	.347	.322	.312	.342	.069					
Q7	5.76	0.99	-0.70	0.59	.265	.259	.232	.247	.102	.533				
Q8	5.48	1.17	-0.60	0.40	.122	.132	.151	.202	.074	.304	.275			
Q9	6.08	0.84	-0.91	1.40	.200	.312	.274	.308	.108	.393	.330	.371		
Q10	5.41	1.15	-0.64	0.75	.262	.341	.263	.324	.131	.360	.373	.269	.301	

Means and standard divisions were calculated using SPSS 22.0. Skewness, kurtosis, and the correlation matrix were derived from AMOS 22.0.

Workers (n = 154)

	M	SD	Skewness	Kurtosis	1.	2.	3.	4.	5.	6.	7.	8.	9.	10.
Q1	5.95	0.91	-0.47	-0.44										
Q2	5.67	0.96	-0.27	-0.68	.545									
Q3	5.68	1.00	-0.37	-0.56	.521	.380								
Q4	5.57	1.22	-0.56	0.01	.386	.263	.357							
Q5	5.27	1.20	-0.29	-0.53	.186	.231	.232	.421						
Q6	5.95	0.89	-0.36	-0.80	.368	.272	.427	.345	.128					
Q7	6.10	0.87	-0.55	-0.65	.336	.203	.376	.430	.319	.616				
Q8	5.46	1.05	-0.29	-0.32	.046	.055	-.003	.100	-.101	.226	.345			
Q9	6.03	0.80	-0.29	-0.84	.412	.360	.593	.296	.160	.524	.370	.191		
Q10	5.20	1.01	0.08	0.05	.344	.189	.343	.230	.084	.439	.357	.232	.329	

Means and standard divisions were calculated using SPSS 22.0. Skewness, kurtosis, and the correlation matrix were derived from AMOS 22.0.

Cancer patients (n = 51)

	M	SD	Skewness	Kurtosis	1.	2.	3.	4.	5.	6.	7.	8.	9.	10.
Q1	5.88	1.48	-1.48	1.82										
Q2	6.16	1.39	-2.26	5.29	.739									
Q3	6.04	1.30	-1.69	3.11	.598	.607								
Q4	5.82	1.45	-1.39	1.51	.754	.668	.535							
Q5	5.76	1.44	-1.05	0.67	.316	.569	.295	.478						
Q6	6.00	1.54	-1.67	2.36	.766	.590	.543	.583	.299					
Q7	6.12	1.34	-2.10	4.34	.787	.625	.679	.609	.286	.799				
Q8	5.86	1.25	-1.85	4.40	.749	.565	.510	.626	.349	.834	.753			
Q9	6.08	1.29	-1.94	4.36	.590	.671	.452	.561	.624	.463	.538	.514		
Q10	5.61	1.64	-1.45	1.63	.575	.502	.516	.517	.232	.548	.570	.579	.468	

Means and standard divisions were calculated using SPSS 22.0. Skewness, kurtosis, and the correlation matrix were derived from AMOS 22.0.
